# Neural Lineage Differentiation From Pluripotent Stem Cells to Mimic Human Brain Tissues

**DOI:** 10.3389/fbioe.2019.00400

**Published:** 2019-12-06

**Authors:** Yean Ju Hong, Jeong Tae Do

**Affiliations:** Department of Stem Cell and Regenerative Biotechnology, KU Institute of Science and Technology, Konkuk University, Seoul, South Korea

**Keywords:** pluripotent stem cell, differentiation, neural, brain, organoid

## Abstract

Recent advances in induced pluripotent stem cell (iPSC) research have turned limitations of prior and current research into possibilities. iPSCs can differentiate into the desired cell types, are easier to obtain than embryonic stem cells (ESCs), and more importantly, in case they are to be used in research on diseases, they can be obtained directly from the patient. With these advantages, differentiation of iPSCs into various cell types has been conducted in the fields of basic development, cell physiology, and cell therapy research. Differentiation of stem cells into nervous cells has been prevalent among all cell types studied. Starting with the monolayer 2D differentiation method where cells were attached to a dish, substantial efforts have been made to better mimic the *in vivo* environment and produce cells grown *in vitro* that closely resemble *in vivo* state cells. Having surpassed the stage of 3D differentiation, we have now reached the stage of creating tissues called organoids that resemble organs, rather than growing simple cells. In this review, we focus on the central nervous system (CNS) and describe the challenges faced in 2D and 3D differentiation research studies and the processes of overcoming them. We also discuss current studies and future perspectives on brain organoid researches.

## Introduction

The ideal scenario for studying human physiology and diseases would be to study cells and tissues directly obtained from the source (e.g., patients). However, human cells or tissues are not easily available and even in cases where they are made available, research with human materials is limited, strictly controlled, and subject to ethical approval from the respective research ethics committees. Compared to other organs, brain tissue is much more difficult to obtain and is also more laborious in the experimental setting. To overcome this issue, immortalized primary human cells have been extensively used. However, immortalized cell lines could never simulate the physiology and function of normal cells in tissues, because of genetic and epigenetic changes such as alterations in gene expression patterns or delayed maturation occurring in the cells during the immortalization process (Farwell et al., 2000). As an alternative approach, animal models have been used to provide the needed tissue or cells. However, there are clear limitations in using animal models to study human disease and physiology. For example, many brain or neurological diseases affecting humans are not present in animals (Nestler and Hyman, 2010; Van der Worp et al., 2010). In addition, humans have complex brain structures distinct from those of other animals, which makes the use of animal models for human brain research unsuitable. The human cerebral cortex is composed of six layers, namely the ventricular zone (VZ), inner (iSVZ) and outer subventricular zone (oSVZ), intermediate zone (IZ), cortical plate (CP), and marginal zone (MZ), whereas the mouse cerebral cortex lacks the oSVZ layer (Zecevic et al., 2005). In addition, the smooth brain of a human fetus later becomes wrinkled to form sulci and gyri which is also not shown in mouse brain (Welker, 1990). Hence, human brain disorders often cannot be explained by the results obtained using animal models. On that note, it is made clear that only human cells should be used to investigate and identify the cause or mechanisms of human neurological diseases. If human cells are to be used, cells capable of both self-renewal and differentiation into specialized cells (i.e., stem cells) during *in vitro* cultivation would be the best option. Adult stem cells recovered from various tissues have a limited self-renewal ability and differentiation potential *in vitro*. On the contrary, pluripotent stem cells can differentiate into every cell type in the body and possess unlimited self-renewal ability, making them a suitable cell type for research. Nowadays, induced pluripotent stem cells (iPSCs) can be easily established from differentiated cells through transduction of reprogramming factors (Takahashi and Yamanaka, 2006; Yu et al., 2007). One of the merits of using iPSCs is the potential to overcome the constraints of limited donor availability and ethical issues associated with human material use (Chun et al., 2010). Thus, many researchers have begun to use iPSCs as a model system for developmental and disease studies, using the differentiation technique and disease modeling, respectively (Saha and Jaenisch, 2009). So far, various cell types, including endodermal, mesodermal, and ectodermal lineages, have been generated from iPSCs and used in disease modeling *in vitro* (Hanna et al., 2007; Raya et al., 2009; Carvajal-Vergara et al., 2010; Liu et al., 2010; Yang et al., 2010; Brennand et al., 2011; Itzhaki et al., 2011; Yazawa et al., 2011). In particular, by differentiating patient-derived iPSCs into a neural lineage, study and modeling on a neurological disease which would otherwise be arduous to perform can be easily conducted. iPSCs have been differentiated into neural stem cells (NSCs) in a 3 dimensional (3D) environment, including neurospheres, and 2 dimensional (2D) NSCs, including rosette-types (Elkabetz et al., 2008) and primitive NSCs (Shin et al., 2019). Continuing efforts on the differentiation technology to mimic brain tissue *in vitro* using pluripotent stem cells have led to technical advances, such as the formation of a mini brain-like structure or brain organoids (Lancaster et al., 2013). In this review, we discuss the technical advances on neural differentiation model systems using pluripotent stem cells toward mimicking the brain tissue and present the obstacles that need be overcome and the future directions in the field.

## 2D Neural Lineage Differentiation

### 2D Neural Lineage Differentiation From Pluripotent Stem Cells

**Figure 1:**
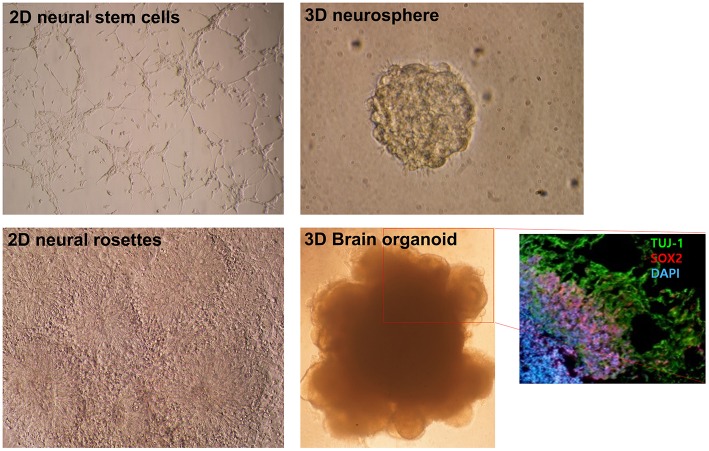
Morphological differences in diverse neural differentiation approaches. The differentiation of pluripotent stem cells into a neural lineage was developed in a stepwise manner: 2D, 3D, and brain organoid. A depiction of the morphologies of growing neural stem cells and neural rosettes in 2D monolayer cultures. A 3D neurosphere formed with the floating culture technique. Folded brain organoid structure formation after culture embedding in Matrigel and differentiation of pluripotent stem cells. Layer division of early neurons (Tuj-1 positive) and neural progenitors (Sox2 positive) identified by immunocytochemistry. This figure was modified with permission from Stem Cell Biology, published by Life Science Publishing Co.

During gastrulation in mammals, the first neural structure that emerges is a form of neural tube consisting of a layer of neuroepithelial cells (Stiles and Jernigan, 2010). Neuroepithelial cells are early neural stem cells that can further differentiate into radial glial cells (RGCs), which are bipolar-shaped neural progenitor cells (NPCs) that can in turn produce both neurons and glial cells, including astrocytes and oligodendrocytes (Malatesta et al., 2000; Noctor et al., 2001; Tamamaki et al., 2001; Merkle et al., 2004). NSCs are tripotent cells that can differentiate into 3 neural lineage cell subtypes: neurons, astrocytes, and oligodendrocytes (Glaser et al., 2007). In addition, NSCs are known to reside in the subventricular zone (SVZ) of the lateral ventricle and subgranular zone (SGZ) of the adult brain hippocampus (Alvarez-Buylla and Lim, 2004). Neural stem cells can be cultured *in vitro* by isolating cells from *in vivo* niches of brain tissues. The “no new neuron hypothesis” was first challenged in 1889 by reports claiming that NSCs capable of producing neuron and glia cells were isolated from an embryonic rat forebrain (Temple, 1989). Since then, isolation of NSCs from the adult central nervous system has been successfully performed in various species of mammals (Reynolds and Weiss, 1992). Both mouse and human NSCs can be isolated and maintained *in vitro* in the presence of extrinsic factors, such as epidermal growth factor (EGF) and fibroblast growth factor 2 (FGF2) (Conti et al., 2005; Figure 1).

Neural lineage differentiation from pluripotent stem cells can be generally achieved under serum-free conditions, which are important for maintaining neural cell cultures. In the past, numerous studies on the differentiation of pluripotent stem cells into nerve cells have been performed, and in this review we focus on representative cases of mouse and human studies (Table 1). The main types of nerve cells that comprise the central nervous system (CNS) are neurons, astrocytes, and oligodendrocytes. A study on the differentiation into each of these cell types and diverse protocols has been previously reported (Cazillis et al., 2006). Retinoic acid (RA) and sonic hedgehog (SHH) were the main factors initially used to drive differentiation of mouse embryonic stem cells (mESCs) into motor neurons (Wichterle et al., 2002). RA has long been known to be a pivotal factor in CNS development and has been used to induce neural differentiation of pluripotent stem cells *in vitro* (Maden, 2007). Members of the Wnt protein family act as a ligand in the signaling pathways activating proliferation and differentiation of NPCs in the CNS (Kuwabara et al., 2009; Inestrosa and Arenas, 2010). A co-culture system was also used to facilitate neural differentiation of mouse (mESCs) and human embryonic stem cells (hESCs). MS5 stromal cells could facilitate neural differentiation induced by the secreted factors, termed stromal cell–derived inducing activity (SDIA) (Bain et al., 1995; Perrier et al., 2004). In neural differentiation of hESCs, the fibroblast growth factor (FGF) functions as both a neural inducing factor and an antagonist to bone morphogenic protein (BMP) (Dhara and Stice, 2008). The still widely used “dual SMAD inhibition” method made use of the Noggin (BMP-antagonist), in conjunction with the SB431542 (TGFβ inhibitor), for the rapid differentiation of hESCs into a neural rosette structure of the early neurectoderm (Chambers et al., 2009). Noggin has been used in neural induction in various species (Lee et al., 2007), while SB431542 has been used for enhancing neural differentiation during embryoid body (EB) formation (Smith et al., 2008). The combination of these 2 SMAD signaling inhibitory factors eliminates the need for an intermediate process, such as EB formation or co-culturing with a stromal feeder, and simplifies the neural induction protocol.

**Table 1:** Neural differentiation approaches in 2D and 3D using stem cells.

**Type**	**Desired cell or tissue type**	**Approaches**	**Characteristics**	**Cell**	**References**
2D	Motor neuron differentiation	• RA, SHH treatment	• Differentiation into spinal progenitor cells and motor neurons • Transplantation of RA and SHH treated EBs into stage 15–17 chick spinal cord	mESCs	Wichterle et al., 2002
	Midbrain DA neuron differentiation	• Stromal cell co-culture • Serum-free condition, LIF removal	• Promoted neural differentiation by SDIA • Anti-neutralizing effect of BMP4	mESCs	Kawasaki et al., 2000
	Midbrain DA neuron differentiation	• Stromal cell co-culture in serum replacement medium • For midbrain DA neuron : N2 medium supplemented with growth factors at various timepoints (SHH, FGF8, BDNF, GDNF, TGFb3, cAMP, and AA)	• Promoted neuroectodermal differentiation by co culture with stromal cells • Efficient midbrain DA neuron derivation	hESCs	Perrier et al., 2004
	Neural precursor cell differentiation	• FGF2 treatment after EB formation	• Formation of neural tube-like structure • Differentiation potential into neurons, astrocytes, and oligodendrocytes • Incorporation of NPCs after transplantation into neonatal mouse brain	hESCs	Zhang et al., 2001
	Neural rosette structure formation Midbrain DA neuron and motor neuron differentiation	• Noggin, SB431542 treatment (Dual-SMAD inhibition) • For midbrain DA neuron : Dual-SMAD inhibition (days 1–5), SHH (days 5–9), BDNF, ascorbic acid, SHH, and FGF8 (days 9–12), BDNF, ascorbic acid, GDNF, TGFb3, and cAMP (days 12–19) • For motor neuron : Dual-SMAD inhibition (days 1–5), BDNF, ascorbic acid, SHH, and RT (days 5–11)	• Conversion of more than 80% of hESCs into neural lineage • Further differentiation into midbrain DA neuron and motor neuron	hESCs	Chambers et al., 2009
	Primitive NSCs (pNSCs) differentiation	• Gibco PSC Neural induction medium	• Efficient induction of pNSCs within 7 days • Expression of NSC marker Pax6, Sox1, Sox2, and Nestin • Differentiation potential to neurons, astrocytes, and oligodendrocytes	hESCs	Yan et al., 2013
	Primitive NSCs (pNSCs) differentiation	• FGF2 and hLIF treatment with GSK inhibitor (CHIR99021) and MEK inhibitor (PD0325901)	• Expression of NSC marker Pax6, Sox1 and N-CAD • Differentiation potential to neurons, astrocytes, and oligodendrocytes	hiPSCs	Shin et al., 2019
3D *in vitro*	NSC proliferation Neural and glial cells differentiation	• 3D peptide scaffold using self-assembly proteins (SAPs)	• Survival and proliferation of NSCs in 3D peptide scaffold • Differentiation potential to neurons, astrocytes, and oligodendrocytes	mNSCs	Cunha et al., 2011
	Transdifferentiation into neuronal or glial cell types	• 3D scaffold synthesized with collagen and hyaluronic acid	• Changes in differentiation potency by scaffold stiffness • Neuronal differentiation in soft scaffold and glial differentiation in stiff scaffold	hMSCs	Her et al., 2013
	Neuronal differentiation	• 3D artificial nanofiber networks	• Rapid and selective differentiation into neurons in artifical nanofiber scaffold	mNPCs	Silva et al., 2004
3D *in vivo*	NSC differentiation	• Teratoma formation	• *In vivo* isolation of NSCs from miPSCs with defected differentiation potency *in vitro* • Expression of NSC marker Nestin, and Sox2 • No secondary tumor formation	mESCs,miPSCs	Hong et al., 2016
	NSC differentiation	• Chimera formation	• Expression of NSC marker Nestin, and Sox2 • More similar gene expression pattern to brain-derived NSCs than *in vitro*-differentiated NSCs	mESCs	Choi et al., 2017

Many researchers, including our group, have tried to generate other types of early-stage neural precursors using mouse or human pluripotent stem cells. Mujtaba et al. (1999) isolated E-NCAM-positive neuroepithelial stem cells from mESCs, similar to fetal neuroepithelial precursors, that were characterized by long-term self-renewal and differentiation potential into neurons and glial cells. Yan et al. (2013) managed to induce hESCs into an earlier NSC cell type, termed primitive NSCs (pNSCs). These pNSCs showed expression patterns similar to those exhibited by the neural rosette and NSCs in the fetal cortex, as well as differentiation potential to neurons, astrocytes, and oligodendrocytes, in addition to the specialized neuronal subtypes, namely GABAergic, dopaminergic, and motor neurons. We have also, recently, succeeded in deriving pNSCs from human iPSCs in the presence of PD0325901 (MEK inhibitor) and leukemia inhibitory factor (LIF) using simple method that does not require EB formation (Shin et al., 2019). Once NSCs are established, the differentiation potential becomes restricted to neurons and glial cells, regardless of NSC type, except in the case of transdifferentiation—a very rare phenomenon—by which a mature cell directly differentiates into different cell lineages (Wagers and Weissman, 2004).

### Advantages and Limitations of 2D Culture Systems

Because NSCs cultured in monolayers mostly comprise a homogeneous population, the 2D culture system can be useful for identification of new drugs that are effective for the treatment of neurological diseases (Pollard et al., 2006). Also, differentiation of patient-derived iPSCs into specific neuronal cell types can be a main concept in cell replacement therapies designed to cure neurodegenerative diseases. For example, human pluripotent stem cell-derived neural cells could alleviate symptoms of neurological disorders when transplanted into animal models of neurological diseases (Perrier et al., 2004; Roy et al., 2006; Kriks et al., 2011). Parkinson's disease (PD) is a neurodegenerative disease characterized by motor dysfunction following the death of dopaminergic (DA) neurons in the midbrain (Davie, 2008). Numerous studies have been conducted to differentiate PSCs into DA neurons to facilitate basic research and for the development of new treatments for PD. Co-culture with MS5 stromal cells was found to increase the efficiency of PSC differentiation into neuroepithelial cells (Perrier et al., 2004). Moreover, these authors found that the treatment with fibroblast growth factor 8 (FGF8) and SHH resulted in high production rate of DA neurons after ventralization (Perrier et al., 2004). Roy et al. co-cultured immortalized human fetal astrocytes with hESCs to improve the their differentiation into DA neurons because astrocytes play an important supporting role in neuronal development (Roy et al., 2006). They observed improvement in motor function after transplantation of these DA neurons into a rat PD model. However, DA neuron transplantation can lead to tumor formation due to the proliferation of undifferentiated neural precursors in the differentiation mixture. To overcome this problem, hPSCs were differentiated into DA neurons via the midbrain floor-plate (FP) precursor where DA neurons are made. After transplantation, the proportion of proliferating cells was reduced to <1% in FP-derived cell grafts and no neural outgrowth was observed; this outcome is comparable to that observed using conventional rosette-derived cell grafts. The FP-derived DA neurons were successfully engrafted with high survival rates, and ameliorated the phenotypic behavior of animal models of PD (Kriks et al., 2011). In addition, to prevent unwanted cell contamination, Doi et al. sorted cells expressing CORIN, an FP marker, which were used for transplantation. For clinical application, they differentiated PSCs in a xeno-free condition using human laminin (Doi et al., 2014). These studies show that the strategy of 2D differentiation into a specific cell type can be safely employed in the treatment of neurological diseases. In addition, many researchers are continuously striving to generate better DA neurons that can mimic there *in vivo* counterparts by using various methods such as co-culture systems, differentiation at specific developmental stages, and using biocompatible materials.

The 2D neural differentiation methods are widely used in the research and preclinical field because they are easy to handle and can utilize various types of NSCs and protocols. Apparently, 2D differentiation methods can be effective in single cell type differentiation (Garavaglia et al., 2010). However, the actual brain does not consist of only one cell type. Azevedo et al. (2009) suggested that the human brain contains ~86 billion neurons and 85 billion non-neuronal cells, including glial cells, immune cells, and endothelial cells. It is also known that more than 10 times more non-neuronal cells populate the brain tissues relative to neurons (Carter, 2014). All brain cells are organically connected and interact with each other. Moreover, it should be heeded that organs are not solely composed of cells. The extracellular matrix (ECM) present among cells in tissues plays important roles in cell adhesion, cell-to-cell communication, cell-to-ECM interaction, cellular differentiation, proliferation, and migration (Theocharis et al., 2016). Since each of the ECM materials bears different characteristics, by culturing cells on the appropriate ECM materials, the stiffness and elasticity of the resulting tissue can be adjusted accordingly. In the central nervous system (CNS), a large proportion (20–30%) of the brain is composed of ECM (Nicholson, 2001; Syková and Nicholson, 2008), the composition of which is known to be unique from other organs. Fibrous proteins, such as laminin, fibronectin, and collagen, are relatively scarce in the brain, whereas proteoglycans and glycoproteins are found in abundance. Therefore, this unique ECM composition may reflect the soft properties exhibited by brain tissue (Ruoslahti, 1996; Novak and Kaye, 2000; Bellail et al., 2004; Hopkins et al., 2015). ECM is also known for its influence on neurite extension and neuronal migration (Snow and Letourneau, 1992; Franco and Müller, 2011). In addition to this haptotaxis property, neurite outgrowth and axon guidance concurrently require chemotactic cues such as nerve growth factor (NGF) concentration gradients (Kapur and Shoichet, 2003). Moreover, NSCs cultured in 2D systems progressively lose the ability to differentiate into neuronal cell types. In conclusion, 2D culture systems cannot mimic the 3D brain environment where cell types are diverse, cells are supported by ECM, and the temporal and spatial concentration gradients of chemical cues are acting in nervous system development. Thus, to study neural development and physiology more closely reflecting the *in vivo* brain system, more advanced culture conditions, other than the 2D differentiation systems, are required. Thus, the need for 3D culture methods has gradually emerged.

## 3D Differentiation Systems

### Creating a 3D Differentiation Environment *in vitro*

Interestingly, the first method used for culturing NSCs *in vitro* was a 3D culture system. Proliferative neural cells isolated from the mouse brain were initially cultured using the neurosphere formation method (Gritti et al., 1996; Figure 1). In order to culture cells in a 3D system, growing cells were supplied with either ECM material or artificial scaffolding (Hopkins et al., 2015). Levenberg et al. (2003) aimed to engineer tissue-like structures using hESCs grown in 3D culture systems, such as biodegradable polymer scaffolds [1:1 mixture of poly L-lactic acid (PLLA) and poly lactic-co-glycolic acid (PLGA)], and observed that the scaffold can affect cellular differentiation and organization (Nseir et al., 2013). Self-assembling peptides (SAPs) have also been used in creating a 3D peptide scaffold shown to support proliferation and differentiation of NSCs (Cunha et al., 2011). In a study using 3D scaffold synthesized with collagen and hyaluronic acid, transdifferentiation of human mesenchymal stem cells (hMSCs) to neuronal and glial cells could be efficiently induced by adjusting the stiffness and porosity of the 3D scaffold (Her et al., 2013). In addition, the topological patterns of the scaffold or biomaterials could also affect proliferation and differentiation of NSCs (Qi et al., 2013; Jeong et al., 2015). When mouse NPCs were cultured within 3D artificial nanofiber networks, neuronal differentiation proceeded rapidly and selectively (Silva et al., 2004). Graphene was suggested to be one of the promising nanomaterials for biological applications, possessing great advantages, such as biocompatibility, flexibility, transparency, and hydrophilicity, and thus when used in culture has led to enhanced neuronal differentiation of hNSCs (Park et al., 2011; Kostarelos and Novoselov, 2014). Although the 3D scaffolds could not support the self-organization of the intrinsic zonal architecture of the tissue, they could provide structural support and enhanced the neural differentiation efficiency. Collectively, the structure, stiffness, porosity, and topological properties of the environment surrounding cells affect not only the characteristics of the cells but also the differentiation direction in the specific lineage (Table 1). In addition to controlling the physical environment, 3D cell culture with control of the hydrodynamic environment has been proposed. Sen et al. suggested that the size of mouse NSC aggregates could be a factor affecting DA neuron differentiation. Therefore, 3D cell culture would allow for obtaining a cell population with uniform size, and for tailoring the size (<150 μm) so as to prevent necrosis of the inner region in large cell aggregates (Sen et al., 2001). In another study, functional DA neurons were generated from neuroepithelial stem cells (NESCs) derived from hiPSCs using a phase-guided 3D cell culture microfluidic bioreactor. These DA neurons expressed tyrosine hydroxylase (TH) with high efficiency (~91%) (Moreno et al., 2015). Thus, providing an *in vitro* 3D environment successfully mimicking the relevant *in vivo* developmental stage could result in the induction of differentiated cells displaying the morphology, characteristics, and functions of that stage, thus simulating natural development as close as possible.

### Neural Differentiation of Pluripotent Stem Cells in a 3D *in vivo* Environment

Although as similar as possible to the *in vivo*-like environment, *in vitro* environment outside the bodies would not be perfect system. To overcome this, one of the alternative options is to induce differentiation of pluripotent cells directly in the *in vivo* environment. Recently, we suggested an *in vivo* neural differentiation method in which pluripotent stem cells were differentiated into NSCs through teratoma formation (Hong et al., 2016; Kim et al., 2017). When pluripotent cells were injected into immunodeficient mice, they formed a teratoma, which is a benign tumor containing cell types of various lineages. Teratoma-isolated Olig2-GFP-positive cells could be established as a pure population of NSCs (Hong et al., 2016). As these established *in vivo* NSCs did not lead to tumor formation when injected back into immunodeficient mice, they were considered safe and applicable to clinical practice. We also suggested another *in vivo* differentiation method, where iPSC-derived NSCs were developed in the brain of chimeric mouse (Choi et al., 2017). First, 13.5 dpc chimeric mouse, which are developed from chimeric blastocyst (formed by iPSCs and morula embryos), were used as *in vivo* NSC forming device. NSCs isolated from the brain of chimeric mouse were closely resembling brain-derived NSCs relative to *in vitro*-differentiated NSCs, as indicated from examination of their gene expression profiles, indicating that *in vivo* differentiation methods can better simulate cells in their vivo environment (Table 1). The *in vivo* NSC derivation system does not require creating an artificial environment, such as ECM, cytokines, and growth factors. However, application of chimeric based NSCs derivation methods in humans seems difficult, because of ethical considerations concerning chimera formations between human and animals and their prohibited status in most countries.

### Organotypic Methods

Along with technical advances in 3D differentiation methods creating an *in vivo*-like environment, “organotypic” method techniques were also developed. An organotypic culture is the culture in which the part of the tissue separated from an organ is cultivated *in vitro* to form organ-like structures. Harrison et al. (1907) cultivated the neural tube of frog embryos in serum using the hanging drop method and observed the obtained neuronal outgrowths. This research demonstrated that cell growth can be possible even if the tissue is removed and grown outside of the body. Thereafter, researchers found that the chick primordia could independently differentiate *in vitro*, leading to self-formation of CNS tissue in its subsequent *in vitro* culture (Hoadley, 1924; Waddington and Cohen, 1936). Another group also showed the *in vitro* self-organization ability of chick and mouse embryo by culturing the dissociated cells acquired from organ rudiments (Moscona and Moscona, 1952; Moscona, 1957). Dissociated cells readily re-aggregated and reconstituted a tissue-like structure. The term “organotypic” was first coined by Reinbold (1954), who observed the ocular differentiation of 3 day chick embryos *in vitro*. Later on, the brain slice culture of vertebrates and the re-aggregation of the chicken brain cell were also studied (Crain, 1966; Ishii, 1966). Organotypic studies were also conducted in other vertebrates such as rats, mice, and humans (Tansley, 1933a,b; Hogue, 1947; Garber and Moscona, 1972a,b; Eugene et al., 2014; Radonjić et al., 2014). In terms of histology, the organotypic culture method can be helpful in observing cell types, relationships, and communication between cells in a particular tissue and has provided the basis for the ongoing organoid research (Shamir and Ewald, 2014; Humpel, 2015). For clinical application, a biopsy-capable tissue may be able to generate organoids based on organotypic culture; however, for some tissue types such as the brain, 3D organoid differentiation will need to be induced using the patient's own iPSCs.

### Self-Assembly Potential of Pluripotent Stem Cells

**Figure 2:**
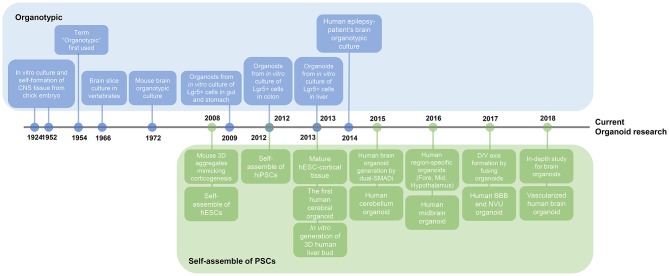
Timeline of key findings in current brain organoid research. Research on organoids began with two studies. Organotypic, a culture method that divides organs or tissues into smaller units and then re-builds them *in vitro*, has long been used for the external growth of tissues. This figure illustrates the presence of adult stem cells in different organs, leading to adult stem cell-based organoid study. On a separate note, the basis for the study of brain organoids has been the plethora of studies of self-organizing pluripotent stem cells. Nowadays, research on brain organoids has surpassed the stage of creating region-specific organoids to better mimic the *in vivo* brain properties, such as vascularization and axis formation.

Prior to the current organoid research, pioneering work in the field had been conducted by the Sasai group (Figure 2). The group established a suspension culture, termed SFEB (serum-free floating culture of embryoid body-like aggregates), that minimized extraneous signals (Watanabe et al., 2005). ESCs cultured in this chemically defined medium without serum were differentiated into neuroectodermal cells, in turn providing cortical progenitors and functional neurons. Notably, they observed that 3D aggregates could mimic embryonic corticogenesis and suggested a self-organization ability of cells during differentiation of hESCs (Eiraku et al., 2008; Mariani et al., 2012). By using this method, the group generated an early-stage self-organized cortical neuroepithelium with immature properties at first, but finally managed to generate more mature hESC-derived cortical tissue that recapitulated second-trimester neocorticogenesis. The generated multi-layered cortical tissue was arranged in the appropriate order, in an inside-out pattern, which is characteristic of the cerebral cortex, and contained an oSVZ layer and outer radial glial cells (oRGCs) which only exist in primates (Kadoshima et al., 2013). The same group also generated an optic-cup structure containing retinal tissue by using 3D differentiation of mESCs and hESCs (Eiraku et al., 2011; Eiraku and Sasai, 2012; Nakano et al., 2012). They suggested that the spontaneous formation of the hemispherical structure in the simple 3D culture might be attributable to the intrinsic self-organizing program of the ESC-derived epithelial structure. This method was later used in generating an eye organoid and identifying the importance of R-spondin in neuroretina differentiation (Takata et al., 2017a). The self-organizing properties of ESC-derived tissues were applicable to generation of other brain regions such as functional adenohypophysis tissue, telencephalic tissue, and polarized cerebellar tissue from hESCs (Suga et al., 2011; Muguruma et al., 2015; Sakaguchi et al., 2015).

## Brain Organoid

### Beginning and Advances in Organoid Research

The word organoid is a compound of the stem words “organ” and “-oid” and refers to the mini organ-like 3D cellular aggregates that resemble actual organ tissues. Cultured organoids include not only tissue-specific cell types but also stem cells that carry self-renewal properties. Hence, organoids can grow by themselves over a long period of time, which is the feature that distinguishes them the most from spheroids. Importantly, organoids contain a variety of cell types that are not merely randomly arranged, but rather preserve the unique intrinsic organization of the desired organ and can mimic *in vivo* organs. Thus, they can recapitulate some functions and developmental details of the specific organ. By culturing organoids that are self-organized *in vitro*, cell-cell interactions between diverse cell types in an organ can be observed. With respect to clinical research and application, organoids have an advantage over 2D culture systems because long-term drug response can be simultaneously evaluated in various cell types, which can accelerate the process of drug discovery.

Initially, organoid research was essentially based on organotypic culture methods (Figure 2). Isolated tissues were used to form aggregates and mini-organ-like structures. In 2009, the Clevers group discovered that leucine-rich repeat-containing G-protein coupled receptor 5 (LGR5) positive gut stem cells could be self-assembled into cellular aggregates and then further grow and form gut epithelial structure *in vitro* termed crypt-villus organoid. When organoids were dissociated into single cells and re-cultured, the dissociated cells could regenerate new organoids, indicating that adult stem cells in organoid are able to continuously organize new tissues (Sato et al., 2009). LGR5, a WNT target gene, is expressed in multipotent adult stem cells in diverse organs, such as intestine, kidneys, stomach, ears, and hair follicles. Hence, researchers have used *Lgr5* as a biomarker of stem cells able to organize and further establish organoids in the stomach, colon, and liver (Sato et al., 2009; Yui et al., 2012; Huch et al., 2013).

An even more promising approach is the generation of organoids using pluripotent stem cells. By using human iPSCs, researchers could avoid the hassle of collecting tissues from human bodies. Acquisition of vascularized and functional human iPSC-derived liver buds (iPSC-LBs) has been described by the Taniguchi group. The group co-cultured human iPSC-derived hepatic cells (iPSC-HEs) with hMSCs and human umbilical vein endothelial cells (HUVECs) based on the idea that organs do not consist of single lineage cells but groups of several tissues. As a result, vascularized and mature liver buds were established by transplantation into immunodeficient mice (Takebe et al., 2013, 2014).

### Brain Organoid

In the case of the brain, and due to the difficulties associated with obtaining tissue from human bodies, brain organoid studies were conducted mainly by using differentiated pluripotent stem cells rather than performing organotypic methods (Figure 2). The first attempt in obtaining a brain organoid was reported in 2013 by the Knoblich group (Lancaster et al., 2013). The group developed a human PSC-derived cerebral organoid culture system by using a Matrigel matrix and a spinning bioreactor. Briefly, the group embedded neural-lineage committed embryoid bodies (EBs) in Matrigel, an artificial ECM, and spin cultured them in bioreactors, enabling EBs to be cultured long-term and mini-brain structure to be form. Today, This method has become one of the standard protocols widely used to generate human brain organoids. This protocol is considered indirect or intrinsic, as it relies on the differentiation potential and the cells' self-organizing program without giving any inductive signals (Lancaster et al., 2013; Lancaster and Knoblich, 2014). This indirect protocol drives the cell fate of the brain organoid into a biased forebrain identity. Nonetheless, the organoid contains various other brain regions, such as the retina, mDA neurons, and even non-ectodermal cells. This great complexity, stochasticity, and self-organization guide the interactions between cells and further promote the maturation of brain organoids (Quadrato et al., 2017). However, this indirect method may produce unwanted regions in the organoid and the pattern of organoid formation is inconsistent in each experiment.

Recently, directed differentiation protocols have also arisen to increase homogeneity of cells that constitute the brain organoid. The “dual-SMAD inhibition” method used for efficient derivation of neurons from pluripotent stem cells, was applied for brain organoid generation (Chambers et al., 2009). This method directed pluripotent stem cells into the neural lineage and inhibited differentiation into other lineages while pre-patterning embryoid bodies. As such, this reduced tissue heterogeneity and increased differentiation efficiency of the neural lineage (Paşca et al., 2015; Qian et al., 2016). After the neural identity of the embryoid body was established using the “dual-SMAD inhibition” method, forebrain organoids were generated in miniaturized spinning bioreactors. In this protocol, the shape and size of the generated forebrain organoids were more consistent relative to those derived by the intrinsic method. Furthermore, the team observed the presence of oRGCs and a well-developed oSVZ-like layer, a specific structure of the developing primate cerebral cortex, in their forebrain organoids. They identified the presence of six cortex layers and subsequent neuronal subtypes in their organoids and observed the growing and maturing cortex through the comparison of organoids of various developmental stages (Qian et al., 2016). Another group also tried to generate midbrain-specific organoids by pre-inducing iPSCs into neuroepithelial stem cells, which are in a more fate-restricted state than pluripotent cells (Monzel et al., 2017). The group produced brain organoids that were highly specific to the midbrain, containing DA neurons with a high degree of neurite myelination by oligodendrocytes. Collectively, the assortment and developmental potential of the starting population of cells directed toward generation of brain organoid could dictate the patterns of the resulting organoids. Since both indirect and direct organoid formation methods have advantages and disadvantages, use of each should be based according to the purpose of the experiment.

### In-depth Analysis of Brain Organoids

Some of the questions arising include, what kind of cells typically reside in a brain organoid, and how similar are brain organoids to the real brain? The brain not only consists of several distinct parts, but each of these parts is composed of and is characterized by diverse sets of cell types. Neurons and glial cells are the major cell types present in the brain, along with endothelial and mesenchymal cells. A large-scale single-cell sequencing of human whole brain organoids has revealed their cellular composition (Quadrato et al., 2017). Six-month-old brain organoids consisted mainly of 7 clusters, one of which was the mesodermal lineage cells. The other 6 clusters were as follows: astroglia, DA neurons, forebrain, retina, neuroepithelial cells, and proliferative progenitors. It was revealed that 3-month-old brain organoids lacked some cell types, such as putative callosal projection neurons, muller glia, and bipolar cells that existed in 6-month-old brain organoids. Moreover, astroglial cells were in a more immature state compared to those in the 6-month-old brain organoids (Quadrato et al., 2017). Recently, the single-cell transcriptome and chromatin accessibility of primate cerebral organoids were investigated (Kanton et al., 2019). Single-cell RNA sequencing analysis revealed that human cerebral organoids generated by an indirect method contained the forebrain, midbrain, hindbrain, retinal cells, and NPCs in the early stage. At the later stage, astrocytes as well as neuronal cell types appeared. Interestingly, human cerebral organoids have been found to develop more slowly than those of chimpanzees and macaques, which corresponds to the difference observed in actual brain development between humans and non-human primates (Kanton et al., 2019). Therefore, these results are indicative of the fact that organoids grown *in vitro* are maturing over time. This notion gives more reason for improving the culture environment when it comes to long-term cultures. Although brain organoids contain neurons and glial cells, their ratio does not usually match the ratio observed in an actual brain. Glial cells play several roles in supporting neurons, and are involved in all aspects of brain function, including nervous system development, nutrition and oxygen supply, elimination of pathogens, as well as synaptic connections. During development, glial cells originate from the radial glial cells, which help in the migration of neurons until 21 weeks gestation in humans, and function as stem cells that ultimately differentiate into neurons and glial cells (Kadhim et al., 1988). Despite the diverse types of neurons investigated in organoid studies, there has been relatively little research performed on glial cells in the CNS and brain organoids. As gliogenesis begins relatively late compared to the development of neurons during development, it might be difficult to identify diverse types of mature glial cells in brain organoids. An in-depth analysis of brain organoids by RNA sequencing revealed that 6-month-old brain organoids contained mature astrocytes (Quadrato et al., 2017). Similarly, another group observed that 8-month-old organoids contained oligodendrocyte progenitors (Renner et al., 2017). Sloan et al. (2017) cultured human cortical spheroids (hCSs) for up to 590 days and conducted single-cell RNA sequencing at multiple time points, which revealed the presence of HepaCAM-positive astrocytes in the CSs at 100 days after culture. These HepaCAM-positive astrocytes showed the morphological and functional properties of mature astrocytes, such as modulation of calcium signaling, synapse formation in neurons, and phagocytic capacity. A study on the differentiation of oligodendrocytes, one of the glial cell types that function in the myelinating neurons, was conducted using the 3D spheroid culture method. To induce oligocortical spheroids from human PSCs, the spheroids were cultured with oligodendrocyte differentiation medium and growth factors, including platelet-derived growth factor AA (PDGF-AA), insulin-like growth factor 1 (IGF-1), and thyroid hormone (T3). Myelination of neurons was observed in 20-week-old oligocortical spheroids, while a maturated state was exhibited in 30-week-old spheroids (Madhavan et al., 2018). Another group that generated “oligodendrocyte spheroids” from human PSCs in a similar manner, identified that that the myelinating oligodendrocytes in 127-day-old spheroids exhibited characteristics similar to those of mature primary oligodendrocytes (Marton et al., 2019). According to the RNA-sequencing results, the spheroids contained oligodendrocytes, neurons, astrocytes, microglia, and endothelial cells. Microglia and endothelial cells are typically hard to find in brain organoids because they are formed from different germ layers and not from the ectoderm (Fatehullah et al., 2016; Dutta and Clevers, 2017). However, a recent report suggested that early-stage cerebral organoids contain microglia that develop from mesenchymal progenitors (Ormel et al., 2018).

In addition to the existence of the oSVZ layer, the presence of brain wrinkles distinguishes the human from the mouse brain. There are numerous wrinkles in the brain of large mammals, such as humans, which were thought to be a measure of intelligence. In fact, wrinkles in the brain widen the entire surface area of the brain, allowing for the processing of more information. However, the human brain folding mechanism was unclear, as it was difficult to observe it live *in vitro*. By using brain organoid technology, a research group was able to observe and analyze the folding process of developing brain organoids. Based on data suggesting that PTEN-AKT signaling controls the cortical formation in the human brain, a study aimed to observe the folding of the cerebral organoid carrying a phosphatase and tensin homolog (PTEN) deletion was conducted (Li et al., 2017). The PTEN mutant brain organoid showed an AKT signaling-induced increase in progenitor proliferation, thereby delaying differentiation and expanding the ventricular zone (VZ) and SVZ regions. Consecutively, the degree of surface folding increased and the surface area widened. Recently, researchers combined physics and biology approaches to analyze the mechanism that drive folding progression and maintenance by using an on-chip approach (Karzbrun et al., 2018).

### Region-Specific Brain Organoids

In addition to the study of whole brain organoids, region-specific brain organoids have been studied, including forebrain, hindbrain, hippocampus, and hypothalamus organoids (Kadoshima et al., 2013; Muguruma et al., 2015; Paşca et al., 2015; Sakaguchi et al., 2015; Ozone et al., 2016; Qian et al., 2016; Shirai et al., 2016). As the brain consists of various regions, researchers have continued to create region-specific brain organoids. Anatomically, the developing brain can be divided in three main regions: forebrain, midbrain, and hindbrain. The forebrain is the largest area of the brain and consists of the cerebrum, thalamus, and hypothalamus. Midbrain acts as a connection between the forebrain and hindbrain and along with hindbrain forms the brainstem. The hindbrain consists of the cerebellum, pons, medulla, and is connected to the spinal cord. In the absence of forced inducing signals, most brain organoids get a forebrain identity by default (Lancaster et al., 2013). To specify hippocampal tissue from hESCs, organoid had to be regulated by BMP and WNT signaling. First, the choroid flexus-like tissue was created by adding a WNT inhibitor (CHIR99021) and BMP4, and then the adjacent tissue medial pallium, a precursor of hippocampus, was generated by reducing the exposure time to these two factors. This medial pallium-like tissue matured into a hippocampal primordium-like tissue expressing markers, such as neuropillin2 (NRP2), and zinc finger and BTB domain containing 20 (ZBTB20). Because of the difficulties associated with long term cultures, tissues were dissociated and attached for an additional 17 wk. After that, hippocampal pyramidal- and granule-like neurons, as well as astrocyte-like cells were identified in the growing aggregates (Sakaguchi et al., 2015). For the generation of hypothalamus specific organoids, pre-patterned by “dual SMAD inhibition” EBs were differentiated into hypothalamic lineages by adding WNT, SHH, and purmorphamine (PMA) followed by a maturation stage with added FGF2 and ciliary neurotropic factor (CNTF). Formed 40-d-old hypothalamic organoids expressed peptidergic neuronal markers and orthopedia (OTP), a marker of the developing hypothalamus (Qian et al., 2016). Midbrain and hindbrain organoids can also be generated by regulating proper signaling pathways required for the developmental process of each region based on research findings from previous brain development and 2D differentiation studies. FGF8 is known to be expressed in the mid-hindbrain boundary (MHB) and plays an important role in the posterior brain region specification while suppressing differentiation into the forebrain lineage (Crossley et al., 1996). By adding FGF8 and SHH as midbrain patterning factors, 3D midbrain-like organoids with functional mDA neurons were successfully generated. These midbrain organoids produced neuromelanin-like granules, which are expressed in the substantia nigra of the midbrain after maturation with several factors (brain-derived neurotrophic factor (BDNF), glial cell line-derived neurotrophic factor (GDNF), ascorbic acid, and db-cAMP) (Jo et al., 2016). Another group also used FGF8, SHH agonists, SMAD inhibitors, and a glycogen synthase kinase 3 beta (GSK3β) inhibitor to generate midbrain-specific organoids (Qian et al., 2016). Midbrain organoids could also be generated from NESCs differentiated from pluripotent stem cells (Monzel et al., 2017). Cerebellum, which controls motor function, is a major part of the hindbrain. Polarized cerebellar tissue could be generated with the caudalizing factor FGF2 at an initial stage followed by the sequential addition of fibroblast growth factor 19 (FGF19) and stromal cell-derived factor 1 (SDF1). This cerebellar tissue had electrophysiologically functional Purkinje neuron cells expressing the maturation markers Purkinje cell protein 2 (L7 or PCP2), Calbindin 1 (CALB1), and LIM homeobox 5 (LHX5). Notably, this hindbrain neural-tube-like tissue gained dorsoventral polarity spontaneously, suggesting FGF19 as the ventralizing factor, since FGF19 treatment led to the increased expression of the ventral markers (Muguruma et al., 2015; Muguruma, 2017). To our knowledge, organoids of other hindbrain structures, such as medulla and pons-specific organoids, have yet to be established.

Region-specific brain organoids are deemed effective in the study of the regional function of local cells, whereas whole-brain organoids are considered much more effective for the systemic study of the overall activity and interactions in the whole of brain. To mimic the actual brain structure, researchers physically combined the individual region-specific organoids after generating each of the desired specific organoids. Organizing the dorsal-ventral axis of the forebrain was performed by 3 individual groups almost at the same time (Bagley et al., 2017; Birey et al., 2017; Xiang et al., 2017). Each group generated dorsal and ventral forebrain organoids separately and combined them to form a whole forebrain, thereby revealing the interneuron migration occurring between the 2 organoids. So as to obtain medial ganglionic eminence (MGE)-like organoids, which correspond to the ventral region, one of the groups, applied a SHH and PMA treatment (Xiang et al., 2017). The 2 other groups used the SAG SHH agonist and the IWP-2 WNT inhibitor as ventral patterning factors (Bagley et al., 2017; Birey et al., 2017). More specifically, Birey et al. (2017) generated a forebrain organoid that could be used as a Timothy syndrome model system, by assembling two individual forebrain spheroids and found that in Timothy syndrome, a neurodevelopmental disorder, interneuron migration between the dorsal and ventral forebrain was defective.

### Vascularization of Brain Organoid

**Figure 3:**
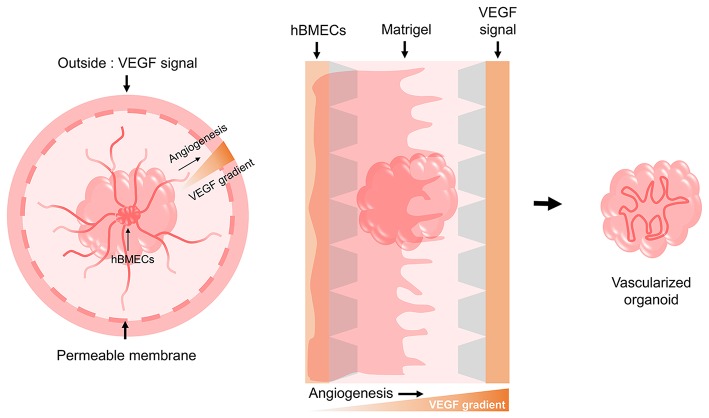
Possible models for the vascularization of brain organoids. Based on the need for a VEGF signal during the angiogenesis of hBMECs that make up the human BBB, we suggest two potential models using culture devices. One model involves implanting the hBMECs inside the brain organoid and then providing the VEGF signal externally through a permeable cell culture dish to induce angiogenesis as it penetrates the brain organoid. The other model involves creating a gradient by providing the VEGF signal to the opposite side of hBMECs using a 3D cell culture chip device, thereby allowing angiogenesis to occur by penetrating the brain organoid.

As the organoids grow bigger over long-term culture, their inner area suffers from a lack of oxygen and nutrient supply. In the body, blood vessels are distributed inside the brain functioning as gas exchange, nutrient exchange, and waste discharge routes, but brain organoids lack these vessel-like structures. Generating brain blood vessels endogenously during the process of brain organoid generation is difficult with the current protocols, but it will be possible in the future to either co-culture cells that can become blood vessels or transplant differentiated blood vessels into brain organoids. Unlike other organs, the cerebrovascular system is characterized by the blood-brain-barrier (BBB) which mainly consists of brain microvascular endothelial cells (BMECs). A number of studies previously conducted aimed to differentiate hPSCs into BMECs. These hPSC-BMECs were sufficiently representative of the endothelial cells present in human BBB and were able to model the BBB phenotype when co-cultured with astrocytes and pericytes (Lippmann et al., 2012; Katt et al., 2016; Hollmann et al., 2017; Qian et al., 2017, 2019; Ribecco-Lutkiewicz et al., 2018). Recently, researchers established a BBB model organoid, a self-assembled spheroid by co-culturing hBMECs or human cerebrovascular microendothelial cells (hCMECs) with astrocytes and pericytes. This BBB organoid was used for testing the permeability of foreign molecules, as it had the ability to regulate transportation of external molecules, which is the most important function of actual BBB (Cho et al., 2017; Bergmann et al., 2018). A vessel composed of BMECs, along with the surrounding astrocytes, pericytes, microglia, ECMs, and neurons are combined to form the basic unit of the brain, the neurovascular unit (NVU). The approach to 3D modeling of the NVU was similarly performed *in vitro*, using a microfluidic system and ECM gels for co-culturing NSCs, BMECs, and MSCs so as to simultaneously induce neurogenesis and angiogenesis (Uwamori et al., 2017). Mansour et al. (2018) reported on obtaining *in vivo* vascularized brain organoids via transplantation into a mouse brain. It should be noted that the extended blood vessels were those of the host and not originating from the transplanted brain organoid. Nevertheless, the meaningful point of this finding is that when they were transplanted, brain organoids tried to survive by sharing the blood vessels of the host. Another group created vascularized brain organoids by embedding the brain organoids and endothelial cells differentiated from iPSCs isolated from the patient into a Matrigel (Pham et al., 2018). In addition, we propose a method for vascularizing brain organoids using hBMECs and culture materials (Figure 3). There are two potentially feasible approaches to vascularize organoids. hBMECs could undergo angiogenesis either inside or outside of the brain organoid. Vascular endothelial growth factor (VEGF) from the cells in organoids may induce the vascularization and migration of hBMECs (Radisavljevic et al., 2000). Vascularization in brain organoids will be important for the long-term culture of larger-sized organoids and is also important for ensuring their similarity to the native brain structure. Importantly, it has shown great promise in cerebrovascular disease research and drug testing.

### Disease Modeling

Beyond basic research on the brain, another goal of the research using organoids may be toward improving human health through the study of diseases. Brain organoid research has facilitated the study of brain diseases by enabling the exploration of the human brain that has not been easily accessible. When combined with research on iPSCs, customized studies can be conducted by establishing patient-specific disease models (Table 2). Microcephaly was the first disease investigated by the Knoblich group using the brain organoid. The group tried to form a brain organoid using skin fibroblast-derived iPSCs from microcephaly patients. However, because microcephaly iPSCs displayed problems in the EB formation stage, the starting point of brain organoid formation, they modified the protocol by increasing the starting cell number. The researchers modified the protocol by increasing the starting cell number. Microcephaly iPSC-derived brain organoids showed a reduced overall size compared to normal brain organoids, especially in the neural progenitor regions due to premature differentiation (Lancaster et al., 2013). Recently, another group attempted to identify the cause of microcephaly in newborns following infection of pregnant women with the ZIKA virus (Garcez et al., 2016). The team infected 3 separate models, a 2D monolayer culture of iPSC-derived NSCs, a neurosphere, and a brain organoid with the ZIKA virus. As a result, the iPSC-derived NSCs and neurosphere models exhibited cell death and disruption, and the brain organoid model showed a 40% decrease in size relative to normal. ZIKA virus preferentially targeted NPCs, therefore early stage organoids suffered the most critical damages, including a reduction in overall size and VZ layer thickness (Qian et al., 2016). The reason for the decrease in NPC pools was that ZIKA virus triggered premature differentiation of NPCs, similar to what was observed in the case of the brain organoids derived from hereditary microcephaly patients (Lancaster et al., 2013; Gabriel et al., 2017). Activation of the Toll-like receptor 3 (TLR3) by ZIKA virus infection was presented as the cause of cell dysregulation and apoptosis, further reinforced by the finding that when TLR3 was inhibited the phenotype was attenuated (Dang et al., 2016). The Miller-Dieker syndrome (MDS) is characterized by abnormally smooth surface and lack of folding structure in the brain (lissencephaly), as well as microcephaly (Dobyns et al., 1983). Due to this odd morphological phenotype, it would be easy to observe its phenotype using a brain organoid. Two groups generated iPSCs-derived cerebral organoids from MDS patients and both of them observed an overall size reduction in the MDS model organoid compared to the wild-type organoid. One study revealed increased apoptosis of NESCs and decreased migration of neurons in the MDS model organoid. The researchers focused on oRGCs and noticed that in the MDS model organoid oRGCs exhibited prolonged mitosis periods (Bershteyn et al., 2017). The second group observed the ventricular zone radial glia cells (vRGCs). In that study, the division plane of vRGCs in the MDS model organoid was changed from symmetric to asymmetric and additionally, the microtubule network organization of these cells was also altered (Iefremova et al., 2017).

**Table 2:** Disease modeling approach using brain organoids.

**Disease model**	**Type of organoid**	**Phenotypes**	**Cell**	**References**
**Neurodevelopmental disease**				
Microcephaly	Forebrain organoid	• Reduction in overall size of organoid • Premature differentiation of NPCs	hiPSCs	Lancaster et al., 2013
Microcephaly (caused by ZIKA virus)	NPCs, Neurosphere brain organoid	• Reduction in overall size of organoid • Cell disruption and death in neurospheres	hiPSCs	Garcez et al., 2016
	Forebrain organoid	• Preferentially infection in NPCs at the early stage organoid • Reduction in overall size and VZ layer thickness	hiPSCs	Qian et al., 2016
	NPCs brain organoid	• Premature differentiation of NPCs • Reduction in VZ and cortical layer • Defect in centrosomal structure of NPCs	hiPSCs	Gabriel et al., 2017
	Cerebral organoid	• Activation of Toll-like receptor 3 (TLR3) after ZIKA infection • Dysregulation of neurogenesis genes • Reduction in overall size and neuroepithelium	hESCs	Dang et al., 2016
Lissencephaly (Miller-Dieker syndrome)	Cerebral organoid	• Reduction in overall size of organoid • Increased apoptosis of NESCs • Decreased migration of neurons • Mitotic delay in oRGCs	hiPSCs	Bershteyn et al., 2017
	Forebrain organoid	• Reduction in overall size of organoid • Division plane of vRGCs was changed from symmetric to asymmetric • Altered microtubule network organization of vRGCs	hiPSCs	Iefremova et al., 2017
Rett syndrome (RTT)	Cerebral organoid	• Increased number of proliferating NPCs • Expanded VZ area • Impaired neurogenesis and maturation	hiPSCs	Mellios et al., 2018
**Neuropsychiatric disease**				
Schizophrenia	Cerebral organoid	• Increased NPC proliferation • Abnormal premature migration of NPCs • Malformation in cortical region	hiPSCs	Stachowiak et al., 2017
**Neurodegenerative disease**				
Alzheimer's disease (AD)	hNPCs embedded in matrigel	• Amyloid-β deposition • Hyperphosphorylation of tau protein • Attenuated symptoms by β- or γ-secretase inhibitor treatment	hNPCs	Choi et al., 2014
	Scaffold-free 3D brain organoid model	• Amyloid-β deposition and hyperphosphorylation of tau protein occurred sequentially over time • Attenuated symptoms by β- or γ-secretase inhibitor treatment	hiPSCs	Raja et al., 2016
	Cerebral organoid	• Amyloid-β deposition • Hyperphosphorylation of tau protein • Similar phenotypes of Down syndrome(DS) brain organoid	hiPSCs	Gonzalez et al., 2018
Parkinson's disease (PD)	Midbrain organoid (Sporadic PD model with LRRK2-muatation)	• Decreased DA neuron and mature neuron • Abnormal localization of α-synuclein • Suggesting a relationship between TXNIP gene and sporadic PD	hiPSCs	Kim et al., 2019

Rett syndrome (RTT) is a neurodevelopmental disease caused by mutations in the X-linked gene methyl-CpG-binding protein 2 (MECP2) (Amir et al., 1999). The effects of MECP2 deficiency in neural development were investigated in both 2D monolayer and 3D brain organoid cultures using RTT patient-derived iPSCs. Along with the result of monolayer differentiation, the RTT model organoid contained an increased number of proliferating NPCs, which resulted in an expanded VZ area, while neurogenesis and neural maturation were impaired (Mellios et al., 2018). Regarding neuropsychiatric diseases, a schizophrenia model organoid exhibited increased proliferation and abnormal premature migration of NPCs, resulting in malformation of the cortical region (Stachowiak et al., 2017).

Alzheimer's disease (AD) is a progressive, age-related neurodegenerative disease causing dementia in late-life (Hardy and Selkoe, 2002). Previously, efforts had been made to model AD through 2D cultures, but there were issues related with the absence of extracellular protein and the diffusion of amyloid-β in the culture medium (D'avanzo et al., 2015). Therefore, researchers applied 3D culture methods to study AD by either Matrigel embedded cultures of hNPCs overexpressing multiple AD mutations, or scaffold-free brain organoid differentiation of iPSCs from AD patients. Both models revealed the common pathology of AD: amyloid-β deposition and hyperphosphorylation of tau protein. These symptoms were attenuated by β- or γ-secretase inhibitor treatment (Choi et al., 2014; Raja et al., 2016). Interestingly, in the scaffold-free 3D model, amyloid-β deposition and hyperphosphorylation of tau protein occurred sequentially as the organoid aged over time (Raja et al., 2016). Another study used iPSCs from AD and Down syndrome (DS) patients to form a cerebral organoid, which also showed the two main pathologies mentioned above (Gonzalez et al., 2018). Parkinson's disease (PD), along with AD, is one of the common neurodegenerative diseases. The pathological feature of PD is degeneration of the mDA neurons, resulting in motor function issues. In addition to the suggestion of PD modeling using a midbrain organoid (Jo et al., 2016), sporadic PD model midbrain organoids from leucine-rich repeat kinase 2 (LRRK2)-mutant iPSCs were recently studied (Kim et al., 2019). This model could reproduce the pathology of PD, demonstrating decreased DA neuron and mature neuron marker expression and abnormal localization of α-synuclein. Furthermore, the group observed upregulation of thioredoxin-interacting protein (TXNIP) expression in the PD model midbrain organoids, whereas when TXNIP was inhibited in knockdown experiments, α-synuclein aggregation was decreased, suggesting a relationship between the TXNIP gene and sporadic PD.

## Conclusion and Future Perspectives

Recent neural differentiation research has focused on the best methods for mimicking the brain tissue-like structure, leading to the development of organoid technology. Despite the remarkable developments in brain organoid research, there remain limitations that require to be overcome. Because the composition of the brain involves complex connections between multiple parts, and region-specific distribution of cells, imitating the structure and function of the whole brain poses an obvious difficulty in brain research. Organoids that simulate the actual cell type ratios and region-specific distribution of cells may be obtained by formulating the optimal cell composition through 3D printing technology. Anterior-posterior and dorsal-ventral axis formation could be induced by establishing concentration gradients of a patterning factor through a microfluidic system or releasable materials. Despite several studies on dorsal-ventral axis formation, all of these attempts focused on generating each part of the brain organoid separately, and then physically merged them together. However, this method was found to be insufficient for observing the complex composition of the brain (Bagley et al., 2017; Birey et al., 2017; Xiang et al., 2017). An alternative approach to mimic the brain axis would be to create concentration gradients of signaling molecules, which might result in an environment similar to that of the developing brain during brain organoid formation. Use of a microfluidic system is one option to produce such spatiotemporal concentration gradients by which complex chemical gradients can be effectively controlled. Park et al. (2009) differentiated hESCs into NPCs by using a microfluidic system to create a gradient of SHH, FGF8, and BMP4. In addition, a signal gradient could be achieved using patterning beads (Kelava and Lancaster, 2016); anterior-posterior axis patterning might be induced by placing the anterior and posterior patterning beads at opposite ends of the organoids. When Takata et al. embedded FGF inhibitor-soaked beads with mESC-derived aggregates, the part adjacent to the beads that received a relatively poor FGF signal developed into the rostral neuroectoderm (Takata et al., 2017b). If these approaches can be employed in a human brain organoid culture system, the composition and axis of the brain organoids could become much more similar to that of the actual brain.

Since human brain is an organ that grows, matures, and changes with learning in the duration of the life of a human, it is realistically difficult for brain research to imitate these changes *in vitro*. Nonetheless, brain organoid studies have gradually started to complete the puzzle of cells present in the brain. Adjusting the proportions and composition of those cells present in each region of the brain will be the next hurdle to be overcome. One of the most urgent issues in the research using brain organoids would be to accelerate the formation of neurons, glial and other support cells in order to better mimic the adult brain. A recent study revealed that cerebral organoids correspond to a second-trimester human fetal brain (Kadoshima et al., 2013). Cerebral organoids represented the similar transcriptional patterns of the early-to-mid human fetal brain and epigenomic signatures of the mid-fetal human brain (Luo et al., 2016). To model brain diseases, acquisition of more mature level of brain organoids is required, as many diseases are undetectable at an early age and do not show phenotypes in infancy, adolescence or even in old age. At present, although brain organoids can be cultured for more than 20 months (Sloan et al., 2017), longer-term cultivation is required to achieve the rate of actual human brain maturation. If it was to exceed 1 year in development, such a model system could not be applied in clinical practice. Thus, a technology that can generate mature brain organoids similar to the adult brain structure in short-term rather than long-term cultures is required. Finally, since the brain and nervous system play significant roles in controlling most of the organs in the body, we expect that further studies on their interactions with other organs using an organ-on-a-chip approach will be a future direction of this field, thereby resulting in new insights for fundamental and clinical applications (Wikswo et al., 2013; Zheng et al., 2016; Zhang et al., 2017).

## Author Contributions

All authors listed have made a substantial, direct and intellectual contribution to the work, and approved it for publication.

### Conflict of Interest

The authors declare that the research was conducted in the absence of any commercial or financial relationships that could be construed as a potential conflict of interest.
